# The use of IT-safety and coping measures against cybercrimes among older adults in Hong Kong: an application of cyber routine activity theory

**DOI:** 10.3389/fsoc.2026.1739787

**Published:** 2026-06-03

**Authors:** Gigi Lam, Bobo Hi-Po Lau, Chung-Kin Tsang, Alex Pak-Ki Kwok, Eric Ngai-Yin Shum

**Affiliations:** 1 Department of Sociology, Hong Kong Shue Yan University, North Point, Hong Kong SAR, China; 2 Department of Counselling and Psychology, Hong Kong Shue Yan University, North Point, Hong Kong SAR, China; 3 Faculty of Social Science, The Chinese University of Hong Kong, Shatin, Hong Kong SAR, China

**Keywords:** Cyber Routine Activity Theory, cybercrimes, guardianship, IT-safety measures, motivated offenders, older adults, scams, suitable targets

## Abstract

**Introduction:**

Older adults are disproportionately vulnerable to cybercrimes such as online and telephone scams. In Hong Kong, the rapid growth in smartphone ownership among adults aged 65 or above has coincided with an increase in cybercrime victimization within this population. However, limited research has examined the specific forms of cybercrimes older adults encounter and how they respond to them in everyday contexts.

**Methods:**

Guided by Cyber Routine Activity Theory (CRAT), this qualitative study explores older adults' experiences of cybercrimes and their coping strategies. Twenty-three focus groups involving 142 adults aged 55 or older were conducted in Hong Kong between May and August 2023. Data was analyzed using deductive and inductive thematic analysis with NVivo 15.

**Findings:**

Findings indicate that impersonation of close relatives, information leakage, and “guess who I am” scams are the most common cybercrimes faced by older adults. Target suitability is shaped by daily smartphone routines and socio-emotional and relational expectations, while motivated offenders employ adaptive, low-cost tactics. Participants relied on both physical and social forms of guardianship, including peer support from individuals with higher digital literacy and the use of social knowledge to reduce scam risks.

**Discussion:**

The study extends CRAT by highlighting the role of socio-emotional commitments rooted in Confucian collectivist culture and by conceptualizing multi-layered and distributed social guardianship. Policy implications at individual, family, and societal levels are discussed.

## Introduction

Population aging has become a global phenomenon, and Hong Kong is no exception. By 2024, the city had become a “super-aged society,” with individuals aged 65 and above accounting for 23.3% of the total population (Census and Statistics Department, 2024). Although a digital divide persists between younger and older generations, smartphone ownership among adults aged 65 or older has risen dramatically, from 68.1% in 2020 to 90.7% and 88% in 2022 and 2024, respectively (Census and Statistics Department, 2023, 2025). This sharp increase can be attributed to two key factors: the government's mandatory smartphone-based track-and-trace policy implemented during the COVID-19 pandemic, and the efforts of organizations that donated smartphones to underprivileged older adults during the pandemic.

As smartphone use among older adults has become more common, the incidence of cybercrimes (i.e., computer-mediated activities which are either illegal or considered illicit by certain parties and which can be conducted through global electronic networks; Thomas and Loader, 2000, p. 3), such as e-shopping fraud, phishing scams, telephone scams, and financial fraud, has also risen. From 2023 to 2024, these types of scams increased by 11.7%, comprising 46.9% of all reported crimes in Hong Kong, despite the overall crime rate rising by only 5% during the same period. Notably, online scams accounted for 61.8% of these cases, highlighting the growing threat of cybercrime (Hong Kong Police Force, 2025).

Globally, the losses to financial scams by the American older adults aged above 60 had experienced an increase of 121% from US$1.4 billion in 2021 to US$3.1 billion in 2022, which then further increased to US$3.4 billion in 2023 (Federal Bureau of Investigation, 2023). In 2024, the top five scams targeting older people in the United States were grandparent scams (scammers pretending to be a younger relative requesting financial assistance), financial services scams (scammers pretending to be a representative of a financial services company), tech support scams (scammers imitating computer troubles by freezing computer or phone screens), government impostor scams (scammers pretending to be a government representative), and romance scams (scammers establishing a fake romantic relationship to obtain money) (U.S. Senate Special Committee on Aging, 2024). Similarly, in Hong Kong, the number of older adult victims of scams rose by 28% from 2023 to 2024 (Ling, 2025). Among these cases, 50% involved online scams, while 40% were related to phone scams, highlighting the growing threat of digital and telecommunication fraud targeting older adults (Ling, 2025).

As older adults may now use smartphone apps more frequently in every aspect of daily life, especially after the pandemic, it is timely to investigate their experiences of online and phone scams. Therefore, the aims of the current study were to (a) examine the types of cybercrimes older adults face when using smart phones and (b) investigate older adults' awareness of IT safety and how they cope with cybercrimes.

### Literature review

While everyone could be susceptible to scams and cybercrimes, older adults face disproportionately heightened risks compared with their younger counterparts (Federal Bureau of Investigation, 2023). The elevated level of victimization among older adults could be understood as the combined outcome of micro level factors, including technical, health, psychological, and social conditions, as well as macro level influences linked to cultural and normative contexts. From a criminological perspective, these factors interact in shaping older adults' exposure, target suitability, and capacity for protection in the digital environment.

#### Micro level factors and cybercrime victimization

At the micro level, prior research indicates that older adults' routine use of smartphones can inadvertently increase their exposure to cybercrime, particularly when limited digital literacy coincides with insufficient protective measures. As smartphones have become essential tools for daily communication, financial transactions, health management, and access to public and private services, older adults increasingly engage in routine online activities such as messaging, clicking links, using social media, and navigating mobile applications (Chen et al., 2025a). Within the cyber routine activity framework, these repetitive and necessary behaviors may position older adults as suitable targets for cyber offenders, particularly in contexts where effective guardianship is weak or absent (Button et al., 2025; Federal Bureau of Investigation, 2023).

Empirical evidence from both Asian and Western contexts consistently shows that older adults with lower levels of digital skills experience significant difficulties in managing privacy settings, creating and maintaining secure passwords, and identifying phishing attempts or fraudulent content. Such limitations are frequently associated with advanced age, lower educational attainment, and reduced levels of social participation (Shin et al., 2025; Zhang et al., 2023). Importantly, these vulnerabilities are not purely technical in nature but are embedded within broader patterns of social inequality, access to resources, and informal support networks.

#### Privacy vulnerabilities, fear of cybercrime, and target suitability

Beyond technical skill deficits, older adults' exposure to scams and cybercrimes is also shaped by a range of age-related psychological, social, and health-related factors. Prior research suggests that privacy vulnerability emerges through the interaction of declining physical and mental health (Deliema et al., 2020; Lee et al., 2021; Li et al., 2016), cognitive changes associated with aging (Burton et al., 2022), low digital literacy (Yang and Jang, 2022), limited social support (Tsai et al., 2017), and reduced social participation (Kim et al., 2017). These conditions jointly limit older adults' capacity to interpret online risks, respond to suspicious interactions, and mobilize protective resources effectively.

Such vulnerabilities are often exacerbated by the lack of age sensitive design in digital interfaces and online services, which frequently fail to accommodate older users' sensory, cognitive, and usability needs (Wang et al., 2019). In addition, fear of cybercrime plays an important role in shaping behavioral responses. Older adults who experience high levels of fear and low confidence in internet use are more likely to withdraw from online activities. While this withdrawal may appear protective, it can paradoxically increase vulnerability by reducing exposure to cybersecurity information and diminishing motivation to seek guidance on safe digital practices (Kemp et al., 2025). Consequently, fear of crime, low self-efficacy, and privacy vulnerabilities may interact to form a self-reinforcing cycle that further increases susceptibility to scam victimization and cybercrime.

#### Macro level cultural contexts and coping with cybercrime

Differences in adaptive and coping strategies are also evident across cultural contexts. In Hong Kong, a survey of 1,061 older adults reported a lifetime fraud exposure rate of 56.1 percent, with phone scams being particularly prevalent at 27.6 percent (Li et al., 2022). Older adults often responded to scams by developing heightened caution driven by fear, relying on community ties to reduce perceived risk, and placing trust in the fairness and responsiveness of the police. At the same time, feelings of shame and concern over loss of face significantly limited formal crime reporting (Li et al., 2022).

In Western societies, coping strategies tend to reflect greater emphasis on individual responsibility and technical self-management. In the United States, older adults have been found to manage privacy risks through selective information disclosure in health applications, often trusting service providers while configuring settings based on prior experience (Zou et al., 2024). Nevertheless, limited digital literacy constrains effective configuration of privacy settings, thereby increasing exposure to cyber risks. In the United Kingdom, older victims of telephone fraud often adopt avoidance strategies such as hanging up calls or blocking numbers, while some engage in formal reporting. However, social isolation can intensify emotional harm, and reporting practices remain inconsistent due to embarrassment and self-blame, mirroring patterns observed in Asian contexts (Button et al., 2025).

These cross-regional patterns reveal uneven adoption of safety practices, persistent gaps in reporting, and divergent reliance on social vs. technical forms of protection. While family support remains central in many Asian contexts, Western approaches rely more heavily on individual technological solutions. This divergence underscores the need for hybrid prevention and intervention strategies that account for cultural norms, social structures, and regional differences.

#### Guardianship and protective responses

To cope with scams and cybercrimes, older adults may choose to rely on either social guardianship or technical guardianship or a combination of both. Social guardianship encompasses assistance provided by family members, peers, and community-based education programs (Tripathi et al., 2019). Such guardianship can take the form of active mitigation strategies, through which social guardians engage older adults in discussions about online content and provide guidance on appropriate internet use (Dedkova and Smahel, 2020; Zou et al., 2024). It may also involve restrictive mitigation strategies, in which guardians monitor or limit online activities by imposing rules or constraints (Brands and van Wilsem, 2021; Lee and Chae, 2012). Technical guardianship includes protective technologies such as antivirus software, two-factor authentication, and other cybersecurity tools designed to reduce exposure to online threats.

Older adults who benefit from stronger forms of social guardianship, particularly support from family members, are generally better at detecting, avoiding, and responding to cyber threats (Chen et al., 2025b). In contrast, insufficient technical guardianship increases target suitability and heightens vulnerability to cybercrime (Chen et al., 2025b). Understanding how older adults perceive, access, and mobilize different forms of guardianship is therefore critical for the development of effective interventions aiming at reducing scam and cybercrime victimization in later life, particularly in the context of Hong Kong's rapidly aging and highly digitalized society.

These fragmented findings point to the need for an integrative theoretical framework to explain how routine smartphone use, guardianship mechanisms, and coping strategies jointly shape cybercrime risk among older adults.

### Theoretical framework

The Routine Activity Theory (RAT) was proposed by sociologists, Lawrence Cohen and Marcus Felson in 1979, as a criminological perspective to explain when, where, and why crime occurs. Rather than focusing on offender dispositions, RAT emphasizes situational factors, including daily routines and environmental or contextual conditions, that facilitate criminal opportunities (Cohen and Felson, 1979). This approach emerged in response to the sociological paradox observed in the United States following the World War II, where crime rates increased despite improved well-being and favorable socioeconomic indicators.

Felson and Cohen (1980) attributed this paradox to structural changes in everyday life, particularly those resulting from increased female labor force participation, rising single-person households, and technological advancement. These changes altered routine activities and reduced guardianship over homes and property, thereby increasing exposure to motivated offenders. Drawing from human ecology theory (Hawley, 1950), which primarily highlighted community structure as a territorial unit, RAT extended the focus to the intersection of victims' and offenders' routine activities across time and space (Cohen and Felson, 1979). In doing so, RAT distinguished itself from other criminological theories prevalent in the late twentieth century that emphasize singular explanations for crime, such as social disorganization theory (i.e., a lack of community cohesion within neighborhoods contributes to higher crime rates), broken windows theory (i.e., tolerating minor offenses encourages more serious and violent crimes), crime pattern theory (i.e., inadequate protection facilitates criminal activity), and crime opportunity theory (i.e., locations with abundant criminal opportunities attract offenders; Ungvarsky, 2024).

RAT posits that a criminal event arises from the convergence of three elements: a motivated offender with the capacity or motivation, a suitable target, and the absence of a capable guardian. This framework is grounded in rational choice assumptions, whereby offenders engage in cost benefit calculations and respond to situational opportunities (Cornish and Clarke, 1986). RAT gained prominence partly due to the limitations of crime prevention programs in the 1970s that focused primarily on offenders' psychological traits while neglecting environmental and situational contexts (Miró, 2014).

Within this framework, a motivated offender refers to any individual with the capacity and inclination to commit a crime. A suitable target may be a person or property characterized by four attributes: value, including both material and symbolic worth; inertia, referring to physical characteristics that may facilitate victimization; visibility, which determines how noticeable the target is to motivated offenders; and accessibility, which reflects how easily the offender can reach the target (Cohen and Felson, 1979). The absence of a capable guardian refers to the lack of any individual or mechanism that could deter criminal activity or assist in crime prevention (Cohen and Felson, 1979).

RAT was originally developed to explain offline predatory crimes such as burglary (Cohen and Felson, 1979), automobile theft (Rice and Csmith, 2002), sexual victimization (Hayes and Maher, 2024), and serial homicide (DeFronzo et al., 2007). The applicability of RAT to cybercrime remains a topic of contentious debate among two groups of criminologists: transformationists and continuists (Leukfeldt and Yar, 2016). Transformationists argue that cyberspace constitutes a novel environment operating within a new spatial dimension that is fundamentally different from the terrestrial world, thereby requiring a distinct analytical framework to examine cybercrime (Capeller, 2001). In contrast, continuists rebut this position. For example, Grabosky (2001) challenges Capeller's overgeneralized claim that virtual and terrestrial crimes are entirely dissimilar. Instead, Grabosky (2001) argues that RAT is applicable to cybercrime in much the same way as it is to street crime, both at the level of individual incidents and in long-term crime trends. This view of homology between cybercrime and terrestrial crime is shared by other continuists (Choo, 2011; McGuire, 2007; Pease, 2001).

Yar (2005) further elaborates on the applicability of RAT to cybercrime by examining its three core elements: motivated offenders, suitable targets, and the absence of capable guardians. While motivated offenders and capable guardianship can largely be treated as homologous across terrestrial and virtual contexts, notable differences emerge in the characteristics of suitable targets, specifically inertia, visibility, and accessibility (derived from the value, inertia, visibility, and accessibility framework, excluding value; Yar, 2005). Yar (2005) and Leukfeldt and Yar (2016) also highlight the spatio-temporally disorganized nature of cybercrime, in which offenders and victims do not necessarily converge at the same time or physical space. For instance, scammers may disseminate fraudulent emails that victims receive and read at a later time. This differs markedly from many offline crimes, which are characterized by spatio-temporal convergence with offenses occurring at a specific time and place (Yar, 2005). Accordingly, Yar (2005) vividly describes cybercrime as “old wine in no bottles” or “old wine in bottles of varying and fluid shape,” in contrast to Grabosky's (2001) depiction of “new wine in old bottles.” Yar (2005) ultimately concludes that RAT concepts can be applied to cybercrime partially, rather than being accepted or rejected wholesale.

Although no definitive conclusion has yet been reached in this debate, a growing body of research has employed RAT as an analytical framework for studying cybercrime (Akdemir and Lawless, 2020; Bossler and Holt, 2009; Chen et al., 2025b; Leukfeldt and Yar, 2016; Marcum et al., 2010; Suzuki et al., 2025). However, its applicability appears to vary across different forms of cybercrime. RAT demonstrates explanatory power for specific offenses, such as identity theft (Reyns et al., 2011; Williams, 2016; van Wilsem, 2013), but is less effective for others. For example, visibility has been shown to influence victimization in hacking, malware infection, identity fraud, consumer fraud, stalking, and threatening communications, whereas value and technical guardianship show little to no effect on cybercrime victimization (Leukfeldt and Yar, 2016). Similarly, guardianship has been found to have no significant association with malware infection (Bossler and Holt, 2009). These mixed findings regarding RAT's applicability to cybercrime have prompted the development of a modified framework, referred to as Cyber Routine Activity Theory (CRAT).

### Cyber routine activity theory

As crime increasingly occurs in the digital environment, RAT has evolved into Cyber Routine Activity Theory (CRAT), which adapts the original framework to the context of cyberspace (Reyns, 2013). Similar to RAT, CRAT emphasizes the interaction among motivated offenders, suitable targets, and the absence of capable guardianship. However, it applies these concepts to online settings that are not constrained by physical time and space (Vakhitova, 2024).

This theoretical evolution reflects two key differences between physical and digital environments, including the spatio-temporal convergence and inadvertent guardianship of the terrestrial crimes. For example, traditional notions of inadvertent guardianship proposed by (Cohen and Felson 1979) are less effective in cyberspace. In online environments, the presence of third-party witnesses rarely increases perceived risk for offenders or deters criminal activity (Vakhitova, 2024). Scholars have argued that guardianship should encompass both intentional and unintentional actions that prevent crime (Hollis-Peel et al., 2011). In cyberspace, personal and technical forms of guardianship and guardianship through active intervention by human third parties are particularly salient and often overshadow traditional inadvertent guardianship (Reyns, 2015).

Cyber guardianship includes several roles. Intimate handlers, such as family members, teachers, and friends, exert social control over offenders through emotional bonds (Felson, 1995). Place managers include individuals or organizations responsible for overseeing digital spaces and reducing target attractiveness by enhancing security and increasing perceived risks (Felson, 1995). Controllers or super controllers refer to individuals or institutions that can prevent or facilitate crime at a broader level (Eck, 1994). Technical guardianship, by contrast, refers to cybersecurity measures such as antivirus software, firewalls, and other digital protection tools.

CRAT has been widely supported by empirical research at both the micro (Akdemir and Lawless, 2020; Kemp, 2023; Ho et al., 2023) and macro levels (Buil-Gil et al., 2021; Chen et al., 2023; Johnson and Nikolovska, 2022) and has proven particularly useful in explaining cybercrime as an outcome of routine online activities shaped by technological advancement (Holt and Bossler, 2014).

By applying CRAT, this study links older adults' everyday smartphone routines with their experiences of cybercrime and their strategies for managing online risks. The theory provides a coherent framework that a) examines the types of cybercrimes older adults face when using smart phones and b) investigates older adults' awareness of IT safety and how they cope with cybercrimes.

## Methodology

### Study design

Twenty-three focus group sessions with a total of 142 older adults aged 55 or older were conducted at three universities in Hong Kong between May and August 2023.

### Participants and recruitment

A purposive sample of community-dwelling Hong Kong adults aged 55 or older who were cognitively sound, Cantonese-speaking, and self-identified as smartphone users was recruited. The participants were recruited through the networks of two local district elderly centers and one local elderly community service organization. Adequacy was ensured through purposeful sampling representing varied demographic and differential experience of using smartphone. Ethical approval was obtained from the Human Research Ethics Committee (HREC) at the affiliated university [HREC 22–05 (M19)]. Informed consent was obtained from the participants prior to their participation.

### Data collection

A pilot focus group lasting for 1 h was conducted with six participants at a neighborhood elderly center in May 2023. Two researchers conducted this pilot focus group, and it was observed by three other researchers. The research team then discussed how to refine the protocol and standardize semi-structured focus group interviews. Ways of engaging each participant within the focus group were also discussed. The focus group protocol consisted of three main components: a) a brief introduction to the use of the internet and apps, b) opinions on IT-related safety measures and scams and c) the use of adaptive strategies related to scams. Data collection comprising 23 focus group sessions with 6–8 participants, each lasting 1–1.5 h, was then carried out by two researchers. After seeking informed consent from the participants, all focus group interviews were audio-recorded. Participants in the focus groups received supermarket coupons with a value of HK$100 as an expression of gratitude.

During data collection, our positionality shaped by our professional backgrounds and the demographic characteristics of the research team had the potential to influence both the conduct and interpretation of the focus groups. The moderator's presence may have introduced unintentional bias through tone, question phrasing, or power dynamics, particularly if participants perceived the moderator as an authority figure. To mitigate these influences, we followed a semi-structured guide, used neutral language, avoided leading questions, engaged in reflexive journaling, and conducted post session debriefings to identify moments when moderator behavior may have affected the discussion. We also employed multiple coders to reduce the impact of individual researcher interpretations and to enhance the credibility and trustworthiness of our findings as shown by informal intercoder reliability in the next section.

### Data analysis

The interview recordings were first manually transcribed in Chinese. Two researchers then carefully read and reread all the transcripts and created an initial codebook based on both deductive and inductive thematic analysis. NVivo 15 was used to conduct the thematic analysis. The first four transcripts were analyzed independently by the other two researchers, and a consensus meeting was then convened to solve any discrepancies and identify any sub-themes. Rather than calculating an intercoder reliability coefficient, we adopted a reflexive, interpretive approach where differences were viewed as productive and informative. Shared interpretive agreement was achieved through discussion among the coding team. After consolidating the codebook by further clustering sub-themes under the main themes, the remaining transcripts were analyzed.

As we conducted 23 focus groups across diverse participant segments, a saturation matrix showed that no new codes or concepts emerged after the 18th focus group. Groups 19–23 produced data that confirmed existing themes without generating novel insights. This indicates that thematic saturation was achieved.

### Findings

#### Profile of the participants

Table 1 presents the profile of the participants. Their ages ranged from 56 to 92 years with a mean age of 69.2 years. The majority of participants were female (71.8%), while the remaining 28.2% were male. Most of the participants completed secondary education (46.5%); 39.4% of the participants lived in public housing, and 35.9% lived in private housing. The majority (66.2%) lived independently from their children.

**Table 1:** Participants' profiles (*n* = 142).

Characteristics	Total (*N =* 142)
Gender, *n* (%)	
Men	40 (28.2)
Women	102 (71.8)
Age (y)	
Mean (SD)	69.2 (5.4)
Median (IQR)	69.5 (73–66)
Education level, *n* (%)	
Primary or below	6 (4.2)
Primary	40 (28.2)
Secondary	66 (46.5)
Post-secondary/University	29 (20.4)
Missing data	1 (0.7)
Living district, *n* (%)	
Kowloon	44 (31.0)
New Territories	34 (23.9)
Hong Kong	3 (2.1)
Missing Data	61 (43.0)
Type of hosing, *n* (%)	
Dormitory	1 (0.7)
Home ownership scheme	32 (22.5)
Private housing	51 (35.9)
Public housing	56 (39.4)
Village house	2 (1.4)
Living with offspring, *n* (%)	
Yes	48 (33.8)
No	94 (66.2)
SNS (e.g., WhatsApp, WeChat, Facebook), *n* (%)	
Never used	1 (0.7)
Seldom used	1 (0.7)
Occasionally used	17 (12.0)
Often used	62 (43.7)
Missing data	61 (43.0)
Online search (e.g., Google), *n* (%)	
Never used	6 (4.2)
Seldom used	16 (11.3)
Occasionally used	15 (10.6)
Often used	44 (31.0)
Missing data	61 (43.0)
Entertainment (e.g., YouTube, Netflix), *n* (%)	
Never used	4 (2.8)
Seldom used	7 (4.9)
Occasionally used	8 (5.6)
Often used	62 (43.7)
Missing data	61 (43.0)
Online shopping (e.g., HKTV mall, Taobao), *n* (%)	
Never used	32 (22.5)
Seldom used	30 (21.1)
Occasionally used	7 (4.9)
Often used	12 (8.5)
Missing data	61 (43.0)
Online banking (e.g., Alipay), *n* (%)	
Never used	35 (24.6)
Seldom used	27 (19.0)
Occasionally used	4 (2.8)
Often used	15 (10.6)
Missing data	61 (43.0)

Regarding digital literacy, while most participants frequently used smartphones for communication (e.g., WhatsApp and WeChat) and for entertainment, a notable proportion had no experience with online shopping (22.5%) or online banking (24.6%).

#### Major themes

The major themes identified were the motivated offenders, older adults as suitable targets and their level of IT safety awareness through examining the presence or absence of capable guardianship at both social and technical levels. The motivated offenders and suitable targets were further divided into sub-themes, including masquerading as close relatives, information leakage, and “guess who I am”.

##### a) Suitable targets and motivated offenders

Older adults are vulnerable to different types of cybercrimes, including the impersonation of close relatives, information leakage, and “guess who I am.” I135, I142, I29, and I8 had received fake phone calls from scammers pretending to be their sons, daughters, or close relatives asking for money.

###### Pretending to be sons and daughters

The following quotes illustrate the impersonation of close relatives in different forms.

“My son does not call me like this. He speaks in Shanghainese with me.” (I135)

“My friend asked the person who they were. The person just called her “mom, mom.” My friend then got angry. She does not have a son, so she just shut down the call.” (I142)

“In 2016, someone told me that my eldest son owed someone money, and I did not know whether I needed to pay the ransom. I just waited, and I needed to find out the truth.” (I29)

“The person pretended to be my son. They had a highly similar voice. I passed the phone to my wife, who also said that the voice was highly similar.” (I8)

###### Guess who I am

Another scam takes the form of asking the recipient to guess the identity of the scammer. I79 and I1 shared their personal experience of “guess who I am” scams.

“The person kept saying “uncle, uncle”... I kept asking ‘who are you?' The person never replied; they just want you to say their identity aloud.” (I79)'

“There are lots of scams. If you know someone, especially the old ones, very few people ask you to guess who they are. That's why they are not your friends if they ask you to guess your identity. Just cut the line. No need to guess anymore.” (I1)

###### Impersonation of government or other organizations

Older adults are also vulnerable to another type of impersonation scam, in which scammers pretend to be from government or other organizations to obtain personal information such as their name and identity card number. I9 and I105 shared their personal experiences.

“I received a phone call which said that I had won a prize in the lucky draw. I replied that I did not even join the lucky draw. The person asked for my name and identity card number. I then replied that if I joined the lucky draw, you must have my name. Then why do I need to give you my identity card number?” (I9)

“I received a call from the government. The government asks me to give personal information … I cut the line because I was afraid my information would be leaked if I stayed on the phone.” (I105)

I63 further explained why older people are vulnerable to being cheated and caught out.

“We lack knowledge. It [how to avoid being scammed] lacks publicity. It has caused our generation to have an avoidance attitude. We want to avoid but we get more afraid. When we are afraid, we are very confused so that we get trapped very easily.” (I63)

##### b) The presence or absence of capable guardianship

To cope with telecommunication scams, the participants had described the presence of both social and technical guardianships.

###### Social guardianship

Social guardianship includes sons and daughters, friends and mass media. Regarding scams impersonating sons or daughters, some participants described checking details with their children.

###### Sons and daughters

“My daughter asks me to contact her. What for? You can be cheated, do you know?” (I30)

“I called my daughter. She asked me why and she asked if someone changed the phone number.” (I136)

“I called my younger son immediately in the club house because I was worried about my eldest son staying in a foreign country. My younger son called his brother immediately to check he was safe.” (I141)

Moreover, the children of older adults asked their parents to call the police.

“My son asked me to call the police. I then went to the police station.” (I29)

Lastly, the older adults described receiving information about apps from younger people.

“The young teach me. When I get back home, they teach me not to do this and download the apps for me. Recently, they installed AVG [free anti-virus software] for me.” (I23)

###### Peers

The second group apart from sons and daughters refers to peers. I13 mentioned the quote as follows.

“I then chatted with my friend. Wow, I am not the only one. Everyone received.” (I13)

Apart from having a discussion with peers, I102 mentioned that they are the social guardians themselves to teach the other peers how to avoid being cheated.

“We are unusual…We teach how to prevent being exploited. We have Senior Police Call [a program initiated by Hong Kong Police Force to enhance community relation and support for the older adults]. We teach and promote how to avoid being cheated.” (I102)

###### Mass media

In addition to peers, I112, I39, I68, and I99 described receiving information from the mass media, including about scam cases, which can enhance awareness about scams and how to prevent them.

“I watched Police Voice and a TV program about scams.” (I112)

“I watch the news and read the newspaper. I know they are scams. I usually cut the line.” (I39)

“I need to thank the publicity on TV [laughing]. I watch a TV program: Scoop. I feel that it is quite good. Lots of publicity … It can really remind you to be more alert.” (I68)

“I tried to borrow other people's phones. The first person refused. The second person also refused and then the third one. The third person said, “Have you watched the TV program Police Voice? You should not lend your phone to others so easily.” (I99)

###### Physical or technical guardianship

Some of the participants opted for physical or technical guardianship to deal with scams, including terminating suspicious calls, installing apps to block suspicious numbers, and checking suspect phone numbers via apps. I15 and I73 stated that they had terminated some unfamiliar calls.

“When I receive calls from the bank or suspicious calls, I cut the line immediately.” (I15)

“This was discussed during smartphone class … They taught us to cut the line on calls we should not receive.” (I73)

I89 and I27 had installed apps to screen the suspect numbers.

“Yes, yes. Installed the screening app “Calls from Little Bear”. (I89)

“I joined the Police Magazine. They have apps. For instance, it you don't trust a phone number, you can visit the apps to check the likelihood of being cheated.” (I27)

The other technical guardianship includes how to use Wi-Fi and password management.

###### Secure Wi-Fi practices

The participants had mixed opinions about Wi-Fi. Some interviewees (I6, I17, and I81) were aware of the added safety of using Wi-Fi at home and questioned the safety of using Wi-Fi in public places.

“The Wi-Fi at home is more secure. For instance, when you conduct a transaction or other private act, you do not know the operators of the Wi-Fi outside … We can refer any problem to the internet service provider. I still stick to my internet service provider when I link to Wi-Fi outside.” (I17)

Similarly, I6 and I81 respectively said that “I will never make bank transactions using Wi-Fi outside” and “I seldom make transactions using Wi-Fi, and we should be very careful when using Wi-Fi outside home.”

Possible reasons why the participants conducted private acts such as online purchase or bank transactions via Wi-Fi at home rather than Wi-Fi outside include leakage of private information and being bombarded with promotional materials.

“When using the Wi-Fi at home, it is not easy for information to leak. It has high security.” (I64)

“When I want to use Wi-Fi during stays at hotels on the mainland, the internet service provider immediately sends me the mainland phone number. The service provider is afraid of information being stolen. It is very common to have a confirmation code on the mainland.” (I72)

“I have tried to connect to Wi-Fi in shopping malls, which was followed by lots of promotions. I asked why. It was because I had used the Wi-Fi before. I then turned off the Wi-Fi and the promotions have never come again.” (I2)

In contrast, some interviewees (I105, I20, I79, I132) said that they only used Wi-Fi outside.

“I just use Wi-Fi for browsing the internet.” (I105)

“It depends on the place. For instance, I log in to Wi-Fi here because it is faster than my limited data. If there is no block, I just log in to Wi-Fi.” (I20)

“Yes, no block. If the Wi-Fi asks for a password, I am blocked. I am more careful about using Wi-Fi in the shopping malls.” (I79)

“I log in to the Wi-Fi at the elderly center. I do not have data at home.” (I132)

###### Password management

The interviewees unanimously agreed about the challenges of remembering and using a password. I36 and I55 described their difficulties as follows.

“The password combination is very complicated with letters and numbers.” (I36)

“I cannot remember passwords correctly. Passwords give me a headache.” (I55)

The inability to remember passwords affected downloading, updating and using apps.

“I get a headache when updating apps with passwords.” (I123)

“I get the apps open, but I don't know how to use them. It requires downloading. I don't know how to do that, and I cannot remember the password. It is troublesome to depend on others.” (I106)

“It requires us to re-enter the password. It creates difficulty for us.” (I21)

Some older adults used active ways to cope with the difficulties of remembering passwords correctly, such as I59, I45, and I7, who described active coping methods.

“I jot down the passwords in my notebook.” (I59)

“When I forget the password, I will ask the young staff at the elderly center.” (I45)

“I went to the supermarket before. Some staff helped with the apps. The staff helped me to set up the apps. The staff asked me to set a password. I then just freely told the staff, but I did not jot down the password. I've already forgotten the password and cannot use the apps anymore.” (I7)

In contrast, I81 and I62 described more passive responses to forgetting passwords:

“I just don't use the apps.” (I81)

“I forget everything. Forget password. Forget username. I can do everything over again.” (I62)

## Discussion

Our findings reveal three dominant types of scams: impersonation of close relatives, impersonation of government or other organizations, and “guess who I am” scams. To cope with these scams, participants mobilized a combination of guardianship that spanned physical or technological guardianship (e.g., screening apps, secure Wi-Fi practices, password management) and social guardianship (e.g., family verification of whereabouts, installation of antivirus software by family members, peer discussion, community support, mass media influence). Contrary to the finding of previous research that older adults tend to adopt avoidance strategies (Frik et al., 2019), our findings suggest that older adults use active strategies, echoing the findings of Zou et al. (2024). Notably, some participants with high levels of digital literacy were social guardians teaching their peers, while some participants also expressed that they leveraged social knowledge to detect scams. Our findings indicate wide variability in the level of digital literacy among older adults. Some of the participants expressed inadequacies in digital literacy, while others were knowledgeable about digital technologies. Such nuances in the “grey digital divide” are in accordance with the findings of a quantitative study conducted by Lau et al. (2025).

These findings collectively demonstrate that cyber victimization and resilience among older adults are shaped by the convergence of routine digital activities, digital literacy, relational dynamics, and all-rounded guardianship. This pattern is well explained by CRAT's emphasis on the situational conditions that facilitate crime events. Regarding the first element of suitable targets, target suitability is closely tied to the level of digital literacy applied during routine activities conducted in familiar encounters (e.g., bank transactions, lucky draws) with familiar individuals (e.g., family members and peers) and in familiar venues (e.g., hotels, supermarkets, shopping malls). This familiarity often makes it difficult for older adults to assess the authenticity of these interactions.

In particular, impersonation and “guess who I am” scams exploit the credibility of family relationships, imitation of familiar voices, interpersonal trust, and authority-based urgency. Scammers thus convert emotional familiarity and relational expectations into tools of manipulation. These findings suggest that for older adults, target suitability is an interaction between daily smartphone routines and social and relational commitment, thereby extending RAT's typical emphasis on embedded socio-emotional commitment within routine activities.

Regarding the motivated offenders, the prevalence of impersonation and “guess who I am” scams reflects the adaptive, low-cost tactics of motivated offenders in mobile ecosystems. Cybercriminals act as motivated offenders who exploit these privacy vulnerabilities through tactics such as fake phone calls, impersonation scams, malicious apps, and fake SMS. They systematically leverage communication platforms where limited contextual cues enable the impersonation of both family members and government or organizational authorities. Their primary objective is to obtain personal information, which can then be used for economic gain, such as demanding ransom in counterfeit kidnapping scenarios or harvesting personal data for resale or secondary fraud (Vakhitova, 2024; Varalakshmi, 2024).

From a CRAT perspective, offenders capitalize on frequent and familiar interactions and the emotional bonding inherent in smartphone use often embedding a sense of urgency to prompt rapid responses in high-risk contexts, such as fabricated kidnappings involving sons or daughters. Another low-cost strategy involves posing as government agencies or other authoritative organizations to pressure older adults into disclosing personal information. Thus, smartphone affordances create opportunity structures that allow motivated offenders to commit with minimal cost and a high degree of convenience and anonymity in the virtual space.

A central contribution of this study is the multi-layered guardianship among older adults. First, physical guardianship was not limited to device settings; participants adopted screening apps, employed link or website vetting tools, practiced Wi-Fi and password management, which is considered an effective way to deter online scams (Cain et al., 2018; Policastro and Payne, 2015). Special attention, however, should be given to the participants' difficulties using passwords, which constitute a form of physical or technical guardianship. The participants' challenges in setting and using passwords were constrained by their digital literacy, in line with the findings of Yang and Jang (2022) and Wang et al. (2025).

Second, social guardianship emerged as distributed and intergenerational: children installed antivirus and verified unusual requests; peers shared warnings, discussed suspicious incidents, and in some cases proactively safeguarded others due to higher digital literacy; and mass media functioned as a super controller by raising awareness and normalizing skepticism. This ensemble of protections shows that, in practice, guardianship is multi-actor and multi-layer, blending human and technological supports that operate before, during, and after exposure to scams, which extended social guardianship beyond family members, peers, and neighbors (Tripathi et al., 2019).

Importantly, some older adults served as peer guardians within their social networks, which extends CRAT beyond a dichotomous concept of presence and absence. Guardianship appears in networked, reciprocal, and dynamic forms: it is cultivated through ongoing social exchange, collective sense-making, and the circulation of security know-how. This suggests that strengthening social infrastructures, such as peer groups, family practices, and media interventions can meaningfully enhance guardianship even when individual technical expertise varies.

Moreover, the prominence of impersonation of government and organizations and relationally framed scams resonates with studies showing that trusting authority is one of the heuristics used by older adults as predicted by the heuristic–systematic model (Shang et al., 2022), which reflects traditional Confucian ideals emphasize filial piety, family harmony, the benevolence of children, and deference to seniority and authorities (Hu et al., 2014).

Where our results add nuance is in the scope and salience of social guardianship. Much cybersecurity guidance centers on individual technical practices (strong passwords, updates, antivirus), but our data shows that collective, relational safeguards (children configuring devices; peers as early-warning systems; mass-media alerts) are equally decisive for older adults' resilience. The importance of social guardianship cannot be studied in isolation from China's collectivist culture, in which family members monitor the online activities of older adults. Understanding family-oriented adaptative strategies as a culture-dependent practice aligns with the collectivist practices reported in other studies conducted in in Hong Kong (Li et al., 2022), China (Chen et al., 2025b) and India (Murthy et al., 2021). The effectiveness of social support in coping with privacy concerns and scams is further bolstered by the social vulnerability model, which attributes fragility to social isolation with social support as a coping buffer (Shang et al., 2022) and highlights the importance of community ties in reducing fear associated with privacy concerns and scams (Li et al., 2022). Research from mainland China indicates that older adults with chronic illnesses often rely on family mediation to cope with online health-related scams. However, in the absence of effective technical guardianship, risky behaviors such as clicking suspicious links frequently persisted, leading to financial loss (Chen et al., 2025b). In these contexts, family members play a central role in managing risk, although this reliance does not always translate into improved technical protection.

In contrast, in Europe, older adults used security software when confident but shifted to avoidance when anxious; self-efficacy enabled actions like updates, whereas worry prompted withdrawal from online activities (Kemp et al., 2025). Collectively, these patterns reveal the uneven adoption of safe practices, with differences in reporting and literacy and family support dominating in Asia while Western strategies lean toward individual tools, indicating a need for hybrid approaches to address cultural and regional differences.

Moreover, the current study document instances where older adults themselves act as guardians for peers and some participants expressed that they use social knowledge to detect scams, referring to knowledge and skills acquired throughout life (Ziegler et al., 2012). Accumulated experience can be independent of technological skill but may incorporate common sense and the experience of established experts (Reeder et al., 2017). This resonates with the findings that older adults have valuable and unique experience and wisdom (Knowles et al., 2021), which challenges portrayals of older adults as a digital marginalized group as postulated by deficit-based narratives (Wild et al., 2008). It also echoes the criticisms upon deficit-based narratives for stereotyping and disempowering all older adults as a vulnerable group (Anaraky and Knijnenburg, 2021; Linton, 2017). It instead highlights contextual integrity (Nissenbaum, 2009) and intra-group heterogeneity (Zou et al., 2024) at both contextual and group and even community levels.

In sum, the current study made theoretical contributions to the CRAT in two following ways. First, we examined target suitability in cyber contexts as an intersection of both routine smartphone activities fueled with relational and emotional dynamic. Incorporating socio-emotional routine into CRAT's conceptualization of target suitability may improve explanatory power for scams that hinge on identity plausibility rather than purely on access or guardianship gaps.

Second, we extend capable guardianship to a multi-layered network of family members, peers, community support and mass media at micro, meso, and macro levels, which constitute multiple and intertwined layers of support across different ecosystems. Guardianship thus operates at multiple scales (dyadic, group, community, societal) and time horizons (preventive literacy, real-time verification, post-incident learning). The importance of both personal and technical guardianship lies in its relationship with target suitability, which refers to the coherence between suitable targets and the presence of capable guardianship within CRAT (Hollis et al., 2013; Vakhitova and Reynald, 2014). In other words, cyber guardianship functions primarily by making the target less suitable, rather than through the mere availability or capability of guardianship itself.

### Policy implications

In addition to the theoretical contributions, the findings of this study have policy implications at the individual, family, and community levels. At the individual level, enhancing awareness of IT safety issues and increasing the anti-scam and digital literacy of older adults are of great importance. These goals can be achieved through digital inclusive classes offered by the government or non-governmental organizations. Technology-savvy older adults can be mentors for their peers or ambassadors who promote IT safety and disseminate information on how to prevent scams, as exemplified by the Smart Seniors Anti-Scam Ambassador Programme introduced by the Hong Kong Monetary Authority and Hong Kong Association of Banks^1^ (The Government of the Hong Kong Special Administrative Region, 2025). Lessons can also be learned from successful anti-scam and online safety campaigns in the United States^2^ (STOP. THINK. CONNECT., 2013), the United Kingdom^3^ (Age UK, 2025), Spain^4^ (Amazon, 2025), and Mexico^5^ (Richardson, 2025). Insights from these campaigns include how to combine outreach, education, community engagement, and cross-sector partnerships (e.g., elderly centers and banks) as well as strengthening intergenerational support with social guardianship.

At the family level, given the effectiveness of social guardianship, how to enhance the awareness and collaboration of social mediators such as close family members is another concern. Sons and daughters should be more concerned about the financial transactions of older adults and maintain a close relationship with their parents. Parents should be reminded to contact their sons and daughters first if they receive a ransom request.

At the community level, given the importance of social support and community ties in collectivist cultures, it is important to seek the collaboration of a range of stakeholders, such as elderly centers and banks. For instance, Hang Seng Bank was the first bank to set up an anti-scam team, which comprises 30 members with banking experience (Pao, 2025). The team members are responsible for giving real-time anti-scam advice to customers, including suggesting that customers stop suspicious transactions and report scams to the police (Pao, 2025). Similarly, 14 banks have implemented interim “Money Safe” measures aimed at protecting and freezing a certain amount of bank deposits for older adults and other customers (The Government of the Hong Kong Special Administrative Region, 2025). Most importantly, increased promotion through TV programs and Police Call should be initiated. For example, the Hong Kong Police Force recently launched an app featuring an AI-chat robot, “Yes Sir,” to answer scam-related enquiries (So, 2025). This new app has been promoted through newspapers, radio, videos, and TV.

## Conclusion

Our findings provide confirmatory evidence for CRAT. First, three types of scams are most prevalent: impersonation of close relatives, impersonation of government or other organizations, and “guess who I am” scams. Second, participants coped with these dominant scam types by employing a combination of physical (or technical) and social guardianship strategies. This study advances CRAT in two important ways. The first contribution is the explanation that cybercrimes arise from the combined influence of digital routines and socio-emotional commitments, along with relational expectations toward family members that are rooted in Confucian collectivist culture. The second major contribution is the expansion and emphasis on multi-layered and distributed social guardianship across different temporal periods, highlighting the need for coordinated efforts at the micro, meso, and macro levels within the digital ecosystem.

Despite these advancements, CRAT remains largely oriented toward situational factors of cybercrime, and little is known about the cognitive-affective processes that shape older adults' reactions under pressure. A valuable direction for future theoretical development is to integrate Protection Motivation Theory (i.e., how individuals cope with fear appeals by considering threat and coping appraisal) into CRAT by examining how threat appraisal and coping appraisal interact with the situational factors and guardianships. Given the rapidly evolving complexity of the digital environment, further research should also investigate the influence of AI and other emerging technologies in conjunction with these theoretical frameworks (Vakhitova, 2024).

The strengths of this study are its large sample size and use of focus groups to gauge the opinions and experience of the participants. Despite these strengths, our investigation is compromised by three limitations. As we only interviewed older adults and did not solicit the opinions and experiences of young and middle-aged adults, we were unable to detect any differences between different age cohorts. Moreover, the dynamics of focus group may prompt socially desirable answers from each other with the specific focus to self-reported coping strategies toward cybercrimes and scams, resulting in perceived competence rather than bona fide effectiveness. In addition, external validity is compromised by research design and sampling with the locally bound study. More attention should be devoted to the exclusion of the most digitally marginalized older adults. A sole inclusion of smartphone users may only give information to the experience of scams and coping strategies by relatively digital savvy older adults. Finally, we did not capture cross-country or regional comparisons. Future research should be oriented toward making comparisons between younger and older adults, and across countries and regions, especially in collectivist and individualistic cultures. Longitudinal research of asking older adults to keep log records is an alternative to current research on self-report measures at a single point of time.

## Data Availability

The raw data supporting the conclusions of this article will be made available by the authors, without undue reservation.

## Footnotes

1 This 2025 campaign sends teams to elderly centers across all 18 Hong Kong districts, providing education and practical anti-scam advice. Older adults are trained to become “anti-scam ambassadors,” promoting awareness within their families and communities. The campaign includes the “Three Anti-Scam Tactics”: Keep Calm, Give Nothing, Verify, and Seek Help. Backed by the banking industry, “Money Safe” protection measures are also publicized for bank deposit security (The Government of the Hong Kong Special Administrative Region, 2025).

2 This ongoing federal campaign partners with organizations to tailor digital safety and scam-prevention resources for older adults. It offers multilingual toolkits, workshops at senior centers, and themed National Internet Safety Months, raising awareness about phishing, technical support scams, fraud, and privacy management (STOP. THINK. CONNECT.™, 2013).

3 Age UK's Scams Prevention and Support Programme is a national program supported by Lloyds Banking Group, which delivers one-to-one advice, group talks, and practical safety training for older adults, with strong community engagement and support for scam victims (Age UK, 2025). Reports indicate substantial improvements in knowledge, confidence, and ability to spot, report, and avoid scams following participation (Age UK, 2025). The key to success in reducing victimization rates and building resilience is to combine education, psychological support, and practical scam detection tools (Age UK, 2025).

4 A scam-prevention campaign introduced by Amazon and 65ymás (the Spanish digital media outlet) distributed educational articles for older consumers and held an expert panel discussion featuring law enforcement, banks, and cybersecurity officials on how to shop safely online. The information provided covered identifying frauds, common scam types, and practical advice for online shopping safety (Amazon, 2025).

5 This program hosts a series of internet safety courses teaching older adults how to avoid online scams, use secure passwords, and improve their general digital skills. These sessions aim to reduce social isolation and empower elders to protect themselves while using new technology (Richardson, 2025).
